# Association of lower extremity range of motion and muscle strength with physical performance of community-dwelling older women

**DOI:** 10.1186/s40101-016-0120-8

**Published:** 2016-12-08

**Authors:** Hungu Jung, Masahiro Yamasaki

**Affiliations:** Graduate School of Integrated Arts and Sciences, Hiroshima University, 1-7-1 Kagamiyama, Higashi-hiroshima City, Hiroshima 739-8521 Japan

**Keywords:** Elderly, Lower extremity, Muscle strength, Performance, Range of motion

## Abstract

**Background:**

Reduced lower extremity range of motion (ROM) and muscle strength are related to functional disability in older adults who cannot perform one or more activities of daily living (ADL) independently. The purpose of this study was to determine which factors of seven lower extremity ROMs and two muscle strengths play dominant roles in the physical performance of community-dwelling older women.

**Methods:**

Ninety-five community-dwelling older women (mean age ± SD, 70.7 ± 4.7 years; age range, 65–83 years) were enrolled in this study. Seven lower extremity ROMs (hip flexion, hip extension, knee flexion, internal and external hip rotation, ankle dorsiflexion, and ankle plantar flexion) and two muscle strengths (knee extension and flexion) were measured. Physical performance tests, including functional reach test (FRT), 5 m gait test, four square step test (FSST), timed up and go test (TUGT), and five times sit-to-stand test (FTSST) were performed.

**Results:**

Stepwise regression models for each of the physical performance tests revealed that hip extension ROM and knee flexion strength were important explanatory variables for FRT, FSST, and FTSST. Furthermore, ankle plantar flexion ROM and knee extension strength were significant explanatory variables for the 5 m gait test and TUGT. However, ankle dorsiflexion ROM was a significant explanatory variable for FRT alone. The amount of variance on stepwise multiple regression for the five physical performance tests ranged from 25 (FSST) to 47% (TUGT).

**Conclusions:**

Hip extension, ankle dorsiflexion, and ankle plantar flexion ROMs, as well as knee extension and flexion strengths may play primary roles in the physical performance of community-dwelling older women. Further studies should assess whether specific intervention programs targeting older women may achieve improvements in lower extremity ROM and muscle strength, and thereby play an important role in the prevention of dependence on daily activities and loss of physical function, particularly focusing on hip extension, ankle dorsiflexion, and ankle plantar flexion ROMs as well as knee extension and flexion strength.

## Background

A previous study [1] involving 6000 participants (aged 5–92 years) showed age-related losses in range of motion (ROM) measurements obtained from seven major joints. Lower extremity ROM is an important predictor of physical function in older adults living in assisted-living housing or skilled nursing facilities [2]. Moreover, decrease in ankle dorsiflexion ROM is significantly smaller in older adults who have suffered a fall than in those who have not [3]. Therefore, the reduction in ROM that occurs with increasing age possibly plays an important role in physical function.

A decline in muscle strength is an important factor impacting the physical function of older adults. Brach and VanSwearingen [4] studied older men who had difficulty managing their activities of daily living (ADL) and found a significant relationship between physical performance test and grip force. A longitudinal study over 2.5 years found that lower quadricep muscle strength was associated with an increased risk of incidents related to mobility limitations in 3075 older adults aged 70–79 years [5]. Moreover, lower knee extension strength in older adults is related to difficulty and disability in performing ADL, such as walking indoors or outdoors [6]. Muscle weakness is associated with poor functional performance and self-reported disability [7].

Performance evaluations have been used to detect functional disability in older adults. The timed up and go test (TUGT) predicts the ability of frail older adults to go out independently [8]. The 5 m gait test, functional reach test (FRT), and five times sit-to-stand test (FTSST) were able to detect mobility limitations in community-dwelling older women, particularly the ability to walk 1/4 mile or climb 10 steps [9]. Furthermore, the four square step test (FSST) was able to differentiate between older adult fallers and non-fallers [10]. Lower extremity performance is related to mobility because mobility difficulties occurring in older adults progress to physical disabilities, whereby they cannot practice ADL independently without assistance [11].

Although women have a consistently larger ROM than men in the same age range [1, 12], age-related decline in ROM is greater in women [13]. In addition, muscle strength is generally weaker in women. In previous studies, hip flexion [2, 14, 15], knee flexion [2, 14, 15], ankle dorsiflexion [2, 14, 16], and ankle plantar flexion ROMs [2, 14, 16] were found to be significantly associated with physical performance, but those studies did not consider hip extension or internal and external hip rotation ROMs. On the other hand, strong knee extension and flexion strength are associated with the maintenance of balance on walking [17]. Although ROM and muscle strength are considered to be important factors that influence physical performance, little information is available on the extent to which each ROM and muscle strength value is associated with the physical performance of community-dwelling older women.

Therefore, the purpose of this study was to determine which of the seven lower extremity ROMs (hip flexion, hip extension, knee flexion, internal and external hip rotations, and ankle dorsiflexion and ankle plantar flexion) and two muscle strengths (knee extension and flexion) play dominant roles in the physical performance of community-dwelling older women.

## Methods

This study was a cross-sectional observational study. Participants were recruited with the assistance of public learning facilities through various channels, including local newspapers, posters, fliers, senior newsletters, visits to tea parties, activities to explain the purpose of the study, and educational seminars regarding physical function, fall prevention, and senior exercise.

The eligibility criteria were as follows: age ≥65 years, living independently in the community, and no serious neurological, musculoskeletal, cognitive, visual, or sensory disorders which would affect their ADL. Individuals who could not perform the physical performance tests or ADL without assistance were excluded from the study. Participants were recruited in Hiroshima, Japan from July 2013 to October 2015. Prior to the beginning of testing, we explained to the participants the purpose and procedures of the study and all participants provided written informed consent. The Ethical Committee of the Graduate School of Integrated Arts and Science of Hiroshima University approved this study (ID: 25–26).

### Procedures

All measurements and self-report questionnaires were conducted in local community centers for ease of accessibility. The five physical performance tests were conducted by either five examiners who took charge of each of the five tests, or by the principal investigator. The five examiners had 2 years of experience in the five physical performance tests and, therefore, were familiar with them. The principal investigator had 5 years of experience in ROM and muscle strength measurements as well as in the five physical performance tests. The participants were instructed that when a test was finished in a section, they should move to another section to be measured for the next test. The participants were able to take a short break between tests. After the physical performance tests, the participants were asked to fill in self-report questionnaires. All self-report questionnaires were checked during face-to-face interviews.

Measurements of ROM and muscle strength were performed on participants 1 week after the physical performance tests. Measurements were taken by the principal investigator with a research assistant; one assisted the participant with the measurement of muscle strength and the other recorded the results. Muscle strength measurements were taken, followed by a 5 min rest, followed by ROM.

### Self-report questionnaires

Age, sex, illness, smoking status, drinking status, pain, and perceived health data were obtained through self-report questionnaires. Body height was measured in centimeters using a vertical standard wall tape. Body mass was measured in kilograms using a calibrated digital scale.

Regarding pain, participants were asked about the presence/absence of pain in the first question, “Have you had any pain in your body during the past 1 month?” Individuals with pain were asked to mark the location of their pain on the McGill pain map (MPM) [18]. The MPM consists of anterior and posterior figures of the body, divided into 36 numbered anatomic regions. The validity and reliability of the MPM have been demonstrated in previous studies　[18, 19]. We investigated pain of the lower extremities, including the lower back, sacrum, hips, buttocks, thighs, knees, legs, ankles, and feet, because we believed that lower extremity pain would affect physical performance. The next question asked was as follows, “Does the pain disturb your daily life activities?” We categorized the lower extremity level of pain as follows: no pain = 0, hardly ever = 1, occasionally = 2, sometimes = 3, frequently = 4, and almost always = 5.

### Lower extremity range of motion

Active ROM was measured using the method specified by Norkin and White [20]. The same principal investigator performed bilateral measurements using a standard goniometer for all lower extremity ROMs to maximize the consistency of the measurement results [15]. In a previous study [21], ROM was measured using the same goniometer by the same examiner for optimal reliability (ICC: 0.80–0.96). Prior to measuring, the participants were not allowed to warm up their body as this may have affected the results. There was a 1 min rest after the measurements were taken in each position. To measure active ROMs, the participants were asked to move the full range of joint motion at a comfortable speed by themselves. Hip extension and knee flexion ROMs were obtained with participants placed in the prone position. Internal and external hip rotation ROMs and ankle dorsiflexion and plantar flexion ROMs were recorded with participants seated on a tall chair with their feet off the floor and knee flexed to 90°. Hip flexion ROM was obtained with participants in the supine position. All ROMs were measured once, and the mean values of the left and right sides of each motion were used for analysis.

### Lower extremity muscle strength

In this study, knee extension and flexion strength measurements were selected based on a previous study [22], which reported that knee strength can be used to characterize overall lower extremity muscle strength. These variables were measured bilaterally by the same principal investigator using a dynamometer (HDD *μ*TasF-1, Anima Corp., Tokyo, Japan). Excellent reliability for muscle strength measurements (ICC: 0.75–0.97) using the same type of dynamometer has previously been reported [23]. The dynamometer was calibrated in a factory prior to measurements. The participants were seated in a constructed chair and were secured by seat belts around his or her body and thigh. Two structures were installed between the chair front legs and between the chair back legs. During the measurements of isometric muscle strength, the dynamometer sensor was fastened to the ankle with Velcro tape, with the trunk and thigh stabilized, while the anchor belt was fixed to an available structure to directly oppose the knee extension and flexion movements. The maximal muscle strength over a 5 s time period was recorded twice and measured in Newtons. There was a 1 min rest between the two trials. The mean values of the two trials on the right and left were used for analysis.

### Physical performance tests

For FRT [24], the participant stood with his/her feet shoulder-width apart and with his/her right arm raised from 90° of flexion along a yardstick placed at the shoulder level and was then asked to reach as far forward as possible, while both feet stayed on the ground to maintain balance. The distance reached was measured in centimeters.

The 5 m gait test [25] was measured as the time taken to walk an 11 m straight line from the first step past the 3 m mark to the first step past the 8 m mark at a comfortable speed.

FSST was performed as previously described [10]. Four squares were placed like a cross on the floor with the tips of four canes facing each other. Canes were approximately 2.5-cm-high and 90-cm-long. The aim of the FSST was to step as fast as possible into each square as follows: the participant stood in square number 1, the test began when the participant stepped forward into square number 2. The participant then stepped clockwise, from square 2 to square 3 moving sideways, backwards to square 4, returning to square 1 moving sideways. Then, stepping counterclockwise, sideways to square 4, forwards to square 3, sideways to square 2, and backwards in square 1 with both feet. The test ended when the participant completed the sequence. The total time taken for FSST was measured.

TUGT [8], modified from an original study, was used in this study. We instructed the participants to rise from an armless chair with a seat height of 43 cm, walk 3 m forward, turn around, return, and sit down. The participants performed TUGT at their usual pace. Timing was calculated from when the participant rose from the initial sitting position at the *go* command to return to sit down.

FTSST [26] was measured as the time taken to stand up and sit down as fast as possible five times from an armless 43-cm-high chair. The test began when the participant stood up from the initial sitting position at the go command and ended when the participant was in the final fully upright position at the end of the fifth stand.

All physical performance tests used in this study involved one practice trial prior to two trial measurements, with a 1 min rest between the two trials. The results of the 5 m gait test, FSST, and FTSST were recorded using a digital stopwatch. Mean values of the two trials were used for further analysis. The FRT [24], 5 m gait test [25], FSST [10], TUGT [8], and FTSST [26] are reliable and valid measures and have previously been reported in the literature.

### Statistical analysis

Pearson correlation coefficients were used to calculate correlations among study variables. Principal component analysis (PCA) was used to identify covariation patterns among seven lower extremity ROMs. To assess the influence of age, BMI, pain, and lower extremity ROM and muscle strength on physical performance, stepwise multiple regression analyses were constructed using the physical performance measures as the dependent variables. Since the five dependent variables were significantly correlated (*r* = −0.30–0.80; *p* < 0.01), a Bonferroni adjustment at a significance level of 0.01 (0.05/5) was used. All data analysis was undertaken on a personal computer using SPSS (version 18, SPSS, JAPAN).

## Results

Characteristics of the 95 participants are shown in Table 1. Apart from 24 participants without pain, most participants had some degree of pain, which disturbed their activities of daily life. Eleven participants were frequently or almost always disturbed by pain.

**Table 1:** Physical characteristics, pain, and perceived health of study population (*n* = 95)

Variables	Mean ± SD
Age (years)	70.7 ± 4.7
Height (cm)	152.4 ± 5.0
Mass (kg)	53.2 ± 6.9
BMI (kg/m^2^)	23.0 ± 3.0
Pain^a^	1.8 ± 1.4
Perceived health (1poor ~ 4very good)	2.9 ± 0.5

^a^No pain = 0. Responses of the subject with pain to the question “Does the pain disturb your daily life activities?” were ranked as follows; hardly ever = 1, occasionally = 2, sometimes = 3, frequently = 4, and almost always = 5

Table 2 shows the illness status, smoking status, and drinking status of participants. Hypertension and arthritis were the most common chronic diseases. Table 3 shows the descriptive statistics for each of the physical performance test measures, the ROMs, and muscle strength measures.

**Table 2:** Illness, smoking, and drinking status

Variables	*n* (%)
Illness	
Cerebrovascular disease	4 (4.2)
Hypertension	30 (31.6)
Osteoporosis	17 (17.9)
Cardiac disease	9 (9.5)
Diabetes	10 (10.5)
Arthritis	30 (31.6)
Pulmonary disease	2 (2.1)
Cancer	9 (9.5)
No illness	29 (30.5)
1 illness	29 (30.5)
2 illnesses	23 (24.2)
3 illnesses	11 (11.6)
4 more than illnesses	3 (3.3)
Smoking	
Current smoker	1 (1.1)
Non-smoker	94 (98.9)
Drinking	
Current drinker	18 (18.9)
Non-drinker	77 (81.1)

**Table 3:** Mean values of physical performance tests, range of motion, and muscle strength

Variables	Mean ± SD	Minimum − maximum
Physical performance tests		
Functional reach Test (cm)	30.1 ± 4.9	18 − 45
5 m gait test (s)	3.3 ± 0.7	2.5 − 6.9
Four square step test (s)	6.7 ± 1.8	4.3 − 17.0
Timed up and go test (s)	6.9 ± 1.5	4.6 − 15.7
Five times sit-to-stand test (s)	7.5 ± 2.0	4.3 − 14.7
Range of motion (degree)		
Hip flexion	122.6 ± 10.8	53 − 144
Hip extension	16.8 ± 4.5	8 − 27
Knee flexion	127.6 ± 8.3	104 − 144
Hip internal rotation	27.9 ± 6.1	14 − 47
Hip external rotation	28.5 ± 4.9	17 − 42
Ankle dorsiflexion	17.7 ± 6.0	5 − 31
Ankle plantar flexion	58.0 ± 7.9	28 − 74
Muscle strength (N)		
Knee extension strength	174.1 ± 53.3	71.8 − 339.5
Knee flexion strength	80.2 ± 27.7	27.0 − 164.5

Pearson correlations between physical performance test scores and participant characteristics are shown in Table 4. Age and pain were significantly associated with all physical performance test scores (*r* = 0.23–0.34; *p* < 0.05).

**Table 4:** Pearson correlations between physical performance test scores and participant characteristics

Characteristic	Functional reach test		5 m gait test		Four square step test		Timed up and go test		Five times sit-to-stand test	
	*r*	*p* value	*r*	*p* value	*r*	*p* value	*r*	*p* value	*r*	*p* value
Age	−0.19	0.063	0.34^b^	<0.001	0.23^a^	0.024	0.36^b^	<0.001	0.24^a^	0.020
Height	0.32^b^	0.002	−0.18	0.074	−0.01	0.891	−0.20	0.052	0.03	0.785
Body mass	−0.18	0.076	0.11	0.292	0.08	0.434	0.08	0.442	0.19	0.060
BMI	−0.34^b^	<0.001	0.20	0.053	0.08	0.439	0.18	0.081	0.18	0.085
Pain	−0.25^a^	0.014	0.23^a^	0.027	0.31^b^	0.002	0.22^a^	0.034	0.26^a^	0.012

^a^ *p* < 0.05

^b^ *p* < 0.01

Table 5 shows the pairwise Pearson correlations among ROMs and muscle strengths. Most of the ROMs and muscle strengths were significantly associated with each other (*r* = 0.20–0.70; *p* < 0.05). The association between hip extension ROM and knee flexion strength was moderate (*r* = 0.42; *p* < 0.001).

**Table 5:** Pairwise Pearson correlations among ranges of motion and muscle strengths

Variables	Hip flexion ROM		Hip extension ROM		Knee flexion ROM		Hip internal rotation ROM		Hip external rotation ROM		Ankle dorsiflexion ROM		Ankle plantar flexion ROM		Knee extension strength		Knee flexion strength	
	*r*	*p* value	*r*	*p* value	*r*	*p* value	*r*	*p* value	*r*	*p* value	*r*	*p* value	*r*	*p* value	*r*	*p* value	*r*	*p* value
Hip flexion ROM			0.36^b^	<0.001	0.45^b^	<0.001	0.34^b^	<0.001	0.20	0.054	0.34^b^	<0.001	0.36^b^	<0.001	0.30^b^	0.003	0.28^b^	0.007
Hip extension ROM					0.42^b^	<0.001	0.37^b^	<0.001	0.35^b^	<0.001	0.31^b^	0.002	0.30^b^	0.003	0.30^b^	0.003	0.42^b^	<0.001
Knee flexion ROM							0.34^b^	<0.001	0.20^a^	0.049	0.39^b^	<0.001	0.22^a^	0.033	0.20	0.050	0.16	0.113
Hip internal rotation ROM									0.15	0.153	0.28^b^	0.007	0.44^b^	<0.001	0.29^b^	0.005	0.24^a^	0.021
Hip external rotation ROM											0.16	0.113	0.15	0.162	0.24^a^	0.018	0.28^b^	0.006
Ankle dorsiflexion ROM													0.21^a^	0.040	0.22^a^	0.033	0.11	0.273
Ankle plantar flexion ROM															0.16	0.130	0.12	0.253
Knee extension strength																	0.70^b^	<0.001
Knee flexion strength																		

*ROM* range of motion

^a^ *p* < 0.05

^b^ *p* < 0.01

**Fig. 1:**
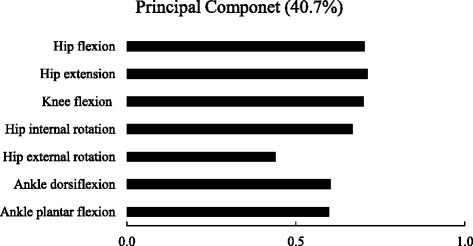
Factor loadings of principal component

PCA extracted only one principal component for ROMs, which explains 40.7% of the variance (eigenvalue = 2.854) (Fig. 1). Extremity positive values of loadings were observed for all ROMs.

Table 6 presents the Pearson correlations of ROM and muscle strength with physical performance test scores. Most ROM and muscle strength variables were consistently associated with physical performance test scores (*r* = −0.21–0.56; *p* < 0.05), particularly hip extension ROM and both types of knee strength which showed a moderate correlation with physical performance test scores (*r* = 0.30–0.56; *p* < 0.01). However, ankle dorsiflexion ROM did not significantly correlate with the 5 m gait test or TUGT.

**Table 6:** Pearson correlations of range of motion and muscle strength with physical performance test scores

Variables	Functional reach test		5 m gait test		Four square step test		Timed up and go test		Five times sit-to-stand test	
	r	*p* value	r	*p* value	*r*	*p* value	*r*	*p* value	*r*	*p* value
Hip flexion ROM	0.32^b^	0.002	−0.29^b^	0.003	−0.20	0.053	−0.32^b^	0.002	−0.26^a^	0.013
Hip extension ROM	0.56^b^	<0.001	−0.33^b^	0.001	−0.41^b^	<0.001	−0.35^b^	<0.001	−0.46^b^	<0.001
Knee flexion ROM	0.45^b^	<0.001	−0.27^b^	0.009	−0.30^b^	0.003	−0.25^a^	0.013	−0.20	0.056
Hip internal rotation ROM	0.25^a^	0.014	−0.32^b^	0.001	−0.25^a^	0.013	−0.38^b^	<0.001	−0.34^b^	<0.001
Hip external rotation ROM	0.38^b^	<0.001	−0.24^a^	0.020	−0.19	0.060	−0.14	0.177	−0.28^b^	0.006
Ankle dorsiflexion ROM	0.45^b^	<0.001	−0.16	0.110	−0.21^a^	0.040	−0.21^a^	0.047	−0.18	0.080
Ankle plantar flexion ROM	0.28^b^	0.006	−0.38^b^	<0.001	−0.17	0.101	−0.51^b^	<0.001	−0.29^b^	0.004
Knee extension strength	0.30^b^	0.003	−0.47^b^	<0.001	−0.42^b^	<0.001	−0.50^b^	<0.001	−0.42^b^	<0.001
Knee flexion strength	0.43^b^	<0.001	−0.42^b^	<0.001	−0.45^b^	<0.001	−0.41^b^	<0.001	−0.48^b^	<0.001

*ROM* range of motion

^a^ *p* < 0.05

^b^ *p* < 0.01

The results of the stepwise multiple regression analyses for each of the physical performance tests are presented in Table 7. Age, BMI, pain, and all lower extremity ROM and muscle strength variables were included in the forward, stepwise conditional regression analyses. Hip extension ROM and knee flexion strength were found to be important explanatory variables for FRT, FSST, and FTSST. In addition, ankle plantar flexion ROM and knee extension strength were significant explanatory variables for the 5 m gait test and TUGT. However, ankle dorsiflexion ROM was a significant explanatory variable for FRT alone. The amount of variance on stepwise multiple regression for the five physical performance tests ranged from 25 (FSST) to 47% (TUGT).

**Table 7:** Stepwise multiple regression analyses using physical performance test scores

Item	Explanatory variable	B	SE	Standardized β	*t*	*p* value	Adjusted *R* ^2^	*F*
Functional reach test	Hip extension ROM	0.32	0.10	0.30	3.16	0.002		
	Ankle dorsiflexion ROM	0.21	0.07	0.26	3.08	0.003		
	Knee flexion strength	0.04	0.02	0.24	2.88	0.005	0.44	19.68
5 m gait test	Ankle plantar flexion ROM	−0.02	0.01	−0.28	−3.25	0.002		
	Knee extension strength	0.00	0.00	−0.38	−4.49	<0.001	0.34	17.15
Four square step test	Hip extension ROM	−0.11	0.04	−0.27	−2.69	0.008		
	Knee flexion strength	−0.02	0.01	−0.34	−3.46	0.001	0.25	16.41
Timed up and go test	Age	0.07	0.03	0.22	2.87	0.005		
	Ankle plantar flexion ROM	−0.08	0.02	−0.41	−5.28	<0.001		
	Knee extension strength	−0.01	0.00	−0.40	−5.27	<0.001	0.47	29.07
Five times sit-to-stand test	Hip extension ROM	−0.14	0.04	−0.31	−3.27	0.001		
	Knee flexion strength	−0.02	0.01	−0.34	−3.60	0.001	0.29	20.35

All regression models were significant (*p* < 0.001)

*ROM* range of motion

## Discussion

We demonstrated that lower extremity ROM and muscle strength were significantly associated with lower extremity physical performance test scores in community-dwelling older women. Only one principal component was extracted by PCA using seven lower extremity ROMs, and most of the lower extremity ROMs were significantly correlated with each other. These results may indicate that lower extremity ROMs, as a whole, were associated with the physical performance of community-dwelling older women. Beissner et al. [2] investigated the relationship between physical performance and ADL and concluded that to maintain the ability to perform ADL, ROM, and muscle strength should be increased in older adults. They also indicated that lower extremity ROM and muscle strength are predictors of functional disability in older adults living in assisted living houses who cannot independently perform for one or more ADL. Consistent with these findings, our results also emphasize the important relationship between lower extremity ROM and muscle strength and physical performance.

We found that hip extension ROM and knee flexion strength were significant explanatory variables for FRT, FSST, and FTSST. It has been reported that restricted hip extension ROM is related to reduced stability of the lumbopelvic region [27], characterized by poor control during a static stabilization task and hip movement while ascending and descending a flight of stairs [28]. Knee flexion strength was poor in participants who had suffered falls because of reduced static and dynamic balance capability [29]. Our results suggested that hip extension ROM and knee flexion strength influence the movements that are required for performing FRT, FSST, and FTSST.

Ankle plantar flexion ROM and knee extension strength were significant determinants of 5 m gait test and TUGT scores. Ankle ROM with reduction in both plantar flexion and dorsiflexion was found to be relevant when performing the figure of 8 walking test (F8WT) [30]. TUGT and F8WT involve similar movements, whereby the participant is required to turn around a cone placed at a distance of approximately 5 ft. Here, ankle plantar flexion ROM was strongly associated with TUGT rather than ankle dorsiflexion ROM. It is likely that ankle plantar flexion ROM largely influences movements during the TUGT compared with ankle dorsiflexion ROM. In addition, these findings are consistent with those of a previous study [31], which reported that knee extension strength was a significant predictor of walking speed. Reduced knee extension strength over a period of 2.5 years in older people was associated with difficulties in walking 1/4 mile without resting [5]. Therefore, it is possible that ankle plantar flexion ROM and knee extension strength are associated with mobility.

Ankle dorsiflexion ROM was a significant explanatory variable only for FRT. A strong association exists between ankle dorsiflexion ROM and FRT in community-dwelling older women [16]. Moreover, ankle dorsiflexion ROM is associated with leaning balance as measured using the maximal balance test in a standing position [31]. These results may indicate that ankle dorsiflexion ROM contributes to the maintenance of an upright posture during forward-reaching tasks.

Our study had several limitations. It is clear that the strength of the associations was relatively modest and that much of the variance in the physical performance tests remained unaccounted for. This may be because our study participants had a high level of perceived health, and there was a relatively weak correlation between pain and physical performance test scores. These characteristics of the sample may have resulted in greater homogeneity than those of previous studies [1, 5]. Furthermore, the results obtained in this study were induced by older Japanese women, and so cannot be generalized for most of the population. It is also possible that other ROM and strength variables should have been evaluated or that the measures selected were too insensitive to detect subtle but significant impairments in ROMs and muscle strength. Ankle inversion-eversion ROM and ankle dorsiflexion strength are significantly correlated with functional performance [16, 26]. However, our study did not conduct ROM and muscle strength measurements for all extremities to minimize the stress of testing on participants.

Despite these limitations, our results have potentially important implications for the physical performance of community-dwelling older women. Considering that associations between lower extremity function, disability, and mortality have been previously reported [32], further investigations on lower extremity ROM and muscle strength for the improvement of physical performance are warranted because such improvement may contribute to the physical functioning of community-dwelling older women.

## Conclusions

We demonstrated that lower extremity ROM and muscle strength were associated with physical performance test results. Hip extension, ankle dorsiflexion, and ankle plantar flexion ROMs, as well as knee extension and flexion strength are important factors that influence the physical performance of community-dwelling older women. Further studies should assess whether specific intervention programs targeting older women may achieve improvements in lower extremity ROM and muscle strength, and thereby play an important role in the prevention of dependence on daily activities and loss of physical function, particularly focusing on hip extension, ankle dorsiflexion, and ankle plantar flexion ROMs as well as knee extension and flexion strength.

## Abbreviations

ADL: Activities of daily living
F8WT: Figure of 8 walking test
FRT: Functional reach test
FSST: Four square step test
FTSST: Five times sit-to-stand test
MPM: McGill pain map
ROM: Range of motion
TUGT: Timed up and go test
